# Role of VEGFR2 in Mediating Endoplasmic Reticulum Stress Under Glucose Deprivation and Determining Cell Death, Oxidative Stress, and Inflammatory Factor Expression

**DOI:** 10.3389/fcell.2021.631413

**Published:** 2021-06-18

**Authors:** Bohan Xu, Linbin Zhou, Qishan Chen, Jianing Zhang, Lijuan Huang, Shasha Wang, Zhimin Ye, Xiangrong Ren, Yu Cai, Lasse Dahl Jensen, Weirong Chen, Xuri Li, Rong Ju

**Affiliations:** ^1^State Key Laboratory of Ophthalmology, Zhongshan Ophthalmic Center, Sun Yat-sen University, Guangzhou, China; ^2^Chengdu Aier Eye Hospital, Chengdu, China; ^3^Division of Cardiovascular Medicine, Department of Medical and Health Sciences, Linköping University, Linköping, Sweden

**Keywords:** eye diseases, visual development, translational vision science, advanced technology, VEGFR2, ER stress, glucose deprivation

## Abstract

Retinal pigment epithelium (RPE), a postmitotic monolayer located between the neuroretina and choroid, supports the retina and is closely associated with vision loss diseases such as age-related macular degeneration (AMD) upon dysfunction. Although environmental stresses are known to play critical roles in AMD pathogenesis and the roles of other stresses have been well investigated, glucose deprivation, which can arise from choriocapillary flow voids, has yet to be fully explored. In this study, we examined the involvement of VEGFR2 in glucose deprivation-mediated cell death and the underlying mechanisms. We found that VEGFR2 levels are a determinant for RPE cell death, a critical factor for dry AMD, under glucose deprivation. RNA sequencing analysis showed that upon VEGFR2 knockdown under glucose starvation, endoplasmic reticulum (ER) stress and unfolded protein response (UPR) are reduced. Consistently, VEGFR2 overexpression increased ER stress under the same condition. Although VEGFR2 was less expressed compared to EGFR1 and c-Met in RPE cells, it could elicit a higher level of ER stress induced by glucose starvation. Finally, downregulated VEGFR2 attenuated the oxidative stress and inflammatory factor expression, two downstream targets of ER stress. Our study, for the first time, has demonstrated a novel role of VEGFR2 in RPE cells under glucose deprivation, thus providing valuable insights into the mechanisms of AMD pathogenesis and suggesting that VEGFR2 might be a potential therapeutic target for AMD prevention, which may impede its progression.

## Introduction

Retinal pigment epithelium (RPE), composed of a monolayer of epithelial cells located between the choroid and retina (Hoon et al., 2014), mediates the exchange of gases, nutrients, and metabolites between the choroid and outer neurosensory retina. Despite multiple functions of RPE in maintaining retinal health and visual function, RPE cells are postmitotic and cannot be replaced by cell division while they become dysfunctional and decline in number with age (Sparrrow et al., 2010). Consequently, ocular diseases such as age-related macular degeneration (AMD), a leading cause of vision loss in developed countries, occurs frequently in the geriatric population (Ambati et al., 2003). Late AMD can be classified into two categories: dry AMD, clinically manifesting as geographic atrophy (GA) and characterized by RPE cell death, and wet or exudative AMD, which features choroidal neovascularization (CNV) penetrating the retina. It has multifactorial causes of pathogenesis, including genetic and environmental factors. Although several mechanisms involving oxidative stress have been investigated (Ambati and Fowler, 2012), pathogenic factors have not been completely inclusive.

Morphological and functional changes in the choriocapillary are strongly associated with AMD. The choriocapillary density was found to be diminished during early AMD (Lee et al., 2018) probably due to endothelial cell loss which occurs prior to RPE degeneration (Biesemeier et al., 2014). Consistently, choroidal blood flow defects have been observed in early/intermediate AMD, which worsened with AMD progression. Choriocapillary flow voids throughout the eye are positively correlated with the rate of geographic atrophy enlargement (Thulliez et al., 2019). Moreover, the CNV region has been found to be environed by a non-perfused choriocapillary region (Treister et al., 2018). These studies suggest that functional defects in choriocapillaris contribute to both dry and wet AMD. Given that the choroid accounts for one of the largest amounts of blood flow in the human body (Lipecz et al., 2019), one can speculate that hypoxia and glucose starvation occur in the RPE at least during certain stages of AMD pathogenesis. Despite the relatively comprehensive studies on hypoxia (Kurihara et al., 2016), much less attention has been paid to the impacts of glucose deprivation on RPE.

Endoplasmic reticulum (ER) stress has been reported to occur in numerous pathological conditions including AMD (Salminen et al., 2010; Kroeger et al., 2019), and excessive ER stress is known to initiate cell-death cascade (Cybulsky, 2017). ER stress is ascribed to an accumulation of misfolded and unfolded proteins in the ER lumen. Theoretically, any factors or conditions that interfere with the folding of nascent proteins in the ER lead to ER stress. These conditions involve ATP depletion, aberration of calcium or redox homeostasis, inhibition of protein transfer to the Golgi apparatus, excessive protein synthesis, or inhibition of protein degradation as well as posttranslational modifications (PTMs) (Kadowaki and Nishitoh, 2019). One of the most important PTMs that ensure that proteins fold correctly is glycosylation (Wang et al., 2015). UDP-N-Acetylglucosamine (UDP-GlcNAc), a required substrate for glycosylation reactions, is generated from glucose via the hexosamine biosynthetic pathway (HBP) (Chiaradonna et al., 2018). Thus, the lack of glucose supply not only reduces ATP production but also affects protein glycosylation and the correct folding in the ER lumen. Unsurprisingly, the lack of glucose triggers ER stress (Badiola et al., 2011).

Endoplasmic reticulum stress activates the unfolded protein response (UPR) primarily via three interconnected signaling pathways, which are initiated by three primary sensors, protein kinase RNA-like endoplasmic reticulum kinase (PERK), activating transcription factor 6α (ATF6α), and inositol-requiring enzyme-1α (IRE1α) (Oakes, 2020). The activation of UPR initially restores ER homeostasis and promotes cell survival by increasing the levels of ER protein-folding enzymes and chaperones, enhancing the degradation of misfolded proteins, and reducing protein translation (Kadowaki and Nishitoh, 2019). When the stress crosses the limit, activation of UPR ultimately triggers cell death. Additionally, accumulating evidence has revealed that ER stress and UPR not only affect the various aspects of proteostasis but also affect the cross-talk with multiple key pathways including inflammation and oxidative stress (Smith, 2018), which link ER stress to many age-related diseases such as AMD (Lipecz et al., 2019). Given that oxidative stress and inflammation play a critical role in cell death and CNV, whether glucose depletion initiates ER stress and further gives rise to cell death and CNV in RPE cells needs to be evaluated.

Currently, even though anti-VEGF antibody based drugs such as ranibizumab, which inhibit CNV, have become a routine treatment for wet AMD treatment (Hernandez-Zimbron et al., 2018), certain clinical studies showed that subretinal fibrosis could occur after anti-VEGF treatment (Daniel et al., 2014). Interestingly, a large fraction of cells consisting of choroidal neovascularization membranes and resulting in subretinal fibrosis were found to originate from RPE cells, indicating that blocking the VEGF-A pathway may change the differentiation status of RPE cells (Hwang et al., 2011). The results from our previous study and other studies have proven that not only RPE cells are the major cell source that secretes VEGF-A but they also express its primary receptor VEGFR2 (Ford et al., 2011; Jia et al., 2017), suggesting the existence of autocrine VEGF-A signaling in RPE cells. Consistently, we reported that VEGFR2 in RPE cells is important for epithelial–mesenchymal transition (EMT) of RPE cells (Du et al., 2018). As a further effort to uncover the roles of VEGF/VEGFR2 signaling in RPE cells, in this study, we examined the involvement of VEGFR2 in glucose deprivation-mediated cell death and the underlying mechanisms. We found that VEGFR2 depletion rescued glucose deprivation-induced cell death, whereas its ligands, either VEGF-A or VEGF-C, were incapable of doing so. RNA sequencing analysis revealed that ER stress and UPR were the most affected pathways, and VEGFR2 depletion significantly decreased the expression of the typical genes related with UPR. The RNA sequencing results were validated by examining individual ER stress markers after knockdown and overexpression of VEGFR2. Mechanistically, VEGFR2-mediated ER stress under glucose starvation in RPE cells most likely occurs owing to the insufficient glycosylation of VEGFR2. Finally, VEGFR2 levels determine the downstream signaling of ER stress including oxidative stress and inflammation. Our study provides insights into the mechanisms of AMD pathogenesis and suggests that VEGFR2 can be used as a novel drug target to prevent AMD initiation or impede the progression of the disease.

## Materials and Methods

### Cell Culture and Treatments

Human RPE cell line ARPE-19 cell was purchased from the American Type Culture Collection (ATCC), and primary human RPE cells (hRPE) were kind gifts from Dr. Hong Ouyang. Both types of cells were cultured in Dulbecco’s modified Eagle’s medium (DMEM) supplemented with 10% fetus bovine serum (FBS), and 100 U/ml streptomycin and penicillin at 37°C with 5% CO_2_ in a humidified incubator. For glucose-deprivation treatment, ARPE-19 or hRPE cells were rinsed with phosphate buffered saline twice and then treated with glucose-free DMEM with or without different percentage of FBS. At different time points as indicated, the cells were trypsinized for cell counting or harvested for western blotting or real-time polymerase chain reaction (rt-PCR). For ER stress inducing experiments, ARPE-19 cells were incubated with 2 μg/ml brefeldin A (BFA, Selleck, #S7046) in DMEM containing 2.5% FBS for 24 h.

### siRNA Transfection and Adenovirus Infection

For gene silencing experiments, Lipofectamine RNAiMAX reagent (Invitrogen, #13778-150) was employed to transfect ARPE-19 or hRPE cells with siRNAs against human VEGFR2, VEGF-A, VEGF-C, c-MET, EGFR1, or control, according to the manufacturer’s instructions. Next, 48 h following transfection, cells were utilized for further treatments as indicated or harvested for western blotting or rt-PCR. All siRNAs were synthesized by RiboBio (RiboBio, China); the siRNA sequences are listed in Table 1.

**TABLE 1:** List of primer sequences used for qPCR.

Gene	Species	Primer sequences (5′ to 3′)
18S	Human	Forward: AGGAATTCCCAGTAAGTGCG
		Reverse: GCCTCACTAAACCATCCAA
VEGFR2	Human	Forward: ATTGGCAGTTGGAGGAAGAG
		Reverse: ATTTCCTCCCTGGAAGTCCT
HMOX1	Human	Forward: GTGCCACCAAGTTCAAGCAG
		Reverse: CAGCTCCTGCAACTCCTCAA
NQO-1	Human	Forward: AGGCTGGTTTGAGCGAGTGT
		Reverse: CCACTCTGAATTGGCCAGAGA
FTL	Human	Forward: ATTTCGACCGCGATGATGTG
		Reverse: CATGGCGTCTGGGGTTTTAC
XBP1s	Human	Forward: GCTCGAATGAGTGAGCTGGA
		Reverse: AGAGGTGCACGTAGTCTGAG
ERN1	Human	Forward: TCTGCAGGCTGCGTCTTTTA
		Reverse: TTCTCATGGCTCGGAGGAGA
ATF6	Human	Forward: ACCTCCTTGTCAGCCCCTAA
		Reverse: GCTCACTCCCTGAGTTCCTG
ATF4	Human	Forward: TCAGTCCCTCCAACAACAGC
		Reverse: CCAACGTGGTCAGAAGGTCA
ERO1α	Human	Forward: TTCTTCGAGCGCCCAGATTT
		Reverse: GCCCAAACCCTGAGTCTGAA
HSPA5	Human	Forward: CTTGCCGTTCAAGGTGGTTG
		Reverse: CCTGACATCTTTGCCCGTCT
DDIT3	Human	Forward: TTCACCACTCTTGACCCTGC
		Reverse: TTCCTGCTTGAGCCGTTCAT
IL6	Human	Forward: CAGCCCTGAGAAAGGAGACAT
		Reverse: TCAGGGGTGGTTATTGCATC
IL8	Human	Forward: GAAACCACCGGAAGGAACCA
		Reverse: ATTTGCTTGAAGTTTCACTGGCA
VEGF-A	Human	Forward: CCTCCGAAACCATGAACTTT
		Reverse: CCACTTCGTGATGATTCTGC
VEGF-B	Human	Forward: TGTCCCTGGAAGAACACAGC
		Reverse: CTGCAGGTGTCTGGGTTGAG
VEGF-C	Human	Forward: GGCTGGCAACATAACAGAGA
		Reverse: GTGGCATGCATTGAGTCTTT
PDGFA	Human	Forward: CGGATACCTCGCCCATGTTC
		Reverse: GCACATGCTTAGTGGCATGG
IL-1β	Human	Forward: CCTGAGCTCGCCAGTGAAAT
		Reverse: TCGTGCACATAAGCCTCGTT
CCL2	Human	Forward: CCTTCATTCCCCAAGGGCTC
		Reverse: GGTTTGCTTGTCCAGGTGGT
BMP2	Human	Forward: TCCTGAGCGAGTTCGAGTTG
		Reverse: TCTCCGGGTTGTTTTCCCAC
INHBA	Human	Forward: AAGAGTGGGGACCAGAAAGAGA
		Reverse: TACCCGTTCTCCCCGACTTT
NRP1	Human	Forward: AGACGGGACCCATTCAGGAT
		Reverse: GCTGATCGTACTCCTCTGGC
ADAM9	Human	Forward: GCACCAAATGTTGGGGTGTG
		Reverse: AGAAGTCCGTCCCTCAATGC
DNAJB9	Human	Forward: TGGCCATGAAGTACCACCCT
		Reverse: AACGCTTCTTGGATCCAGTGT
ERO1B	Human	Forward: AGAGAACTGTTTCAAGCCTCG
		Reverse: TAGGTCCCCAACTGGGCTTA
TMB1M6	Human	Forward: AGGCGGGTTAGGAAGAGTGGA
		Reverse: CAGCCGCCACAAACATACAAA
LMAN1	Human	Forward: CGAGTGACTGGAAGAGGTCG
		Reverse: AAAGCTTGACTAGCCCCGTC
TIMP3	Human	Forward: ACCGAGGCTTCACCAAGATG
		Reverse: CAGGGGTCTGTGGCATTGAT
ICAM1	Human	Forward: ACCATCTACAGCTTTCCGGC
		Reverse: CAATCCCTCTCGTCCAGTCG
YBX3	Human	Forward: GTGCAGAAGCTGCCAATGTG
		Reverse: CTCCTCCTCCCCAGCGTAAT
MET	Human	Forward: TGGTGGAAAGAACCTCTCAACA
		Reverse: GCGATGTTGACATGCCACTG
EGFR	Human	Forward: TGCCACAACCAGTGTGCTG
		Reverse: TGACCATGTTGCTTGGTCCT

For VEGFR2 overexpression, Ad-VEGFR2 adenovirus (Vigene Biosciences, China) carrying the VEGFR2 gene (Ad-VEGFR2 group) was used to infect ARPE-19 cells, while Ad-GFP served as a control group. Next, 48 h following infection, cells were utilized for further treatments as indicated or harvested for western blotting or RT-PCR.

### RNA Sequencing

The primary hRPE cells were transfected with control siRNA and VEGFR2 siRNA followed by culturing under glucose deprivation for 24 h (3 replicates/group). The total RNA was isolated from cells using an RNeasy mini kit (Qiagen, Germany). Paired-end libraries were synthesized using the TruSeq^®^ RNA Sample Preparation Kit (Illumina, United States) following TruSeq^®^ RNA Sample Preparation Guide. Briefly, poly-A containing mRNA molecules were purified using poly-T oligo-attached magnetic beads. Purified libraries were quantified using Qubit^®^ 2.0 Fluorometer (Life Technologies, United States) and validated by Agilent 2100 bioanalyzer (Agilent Technologies, United States) to confirm the insert size and calculate the mole concentration. Clusters were generated by cBot with the library diluted to 10 pM and then were sequenced on the Illumina HiSeq X-ten (Illumina, United States). Library construction and sequencing were performed at Shanghai Biotechnology Corporation. The RNA sequencing data were analyzed in a standard manner. rt-PCR was used to confirm the genes the expressions of which were significantly changed in the results of RNA sequencing analysis to prove its credibility.

Sequenced raw reads were preprocessed by filtering out rRNA reads, sequencing adapters, short-fragment reads, and other low-quality reads. We used Hisat2 (version 2.0.4) to map the cleaned reads to the human GRCh38 reference genome with two mismatches. After genome mapping, Stringtie (version 1.3.0) was run with a reference annotation to generate FPKM values for known gene models. Differentially expressed genes were identified using edgeR. The *P*-value significance threshold for multiple tests was set by the adjusted false discovery rate (FDR) *P*-value using the Benjamini-Hochberg (BH) method. The fold-changes were also estimated according to the FPKM in each sample. The differentially expressed genes were selected using the following filter criteria: FDR ≤ 0.25 and fold-change ≥1.5. ClueGO (version 2.5.3) (PMID: 19237447) was used to interpret the biological significance of key changed genes with Gene ontology (GO) terms and KEGG pathways.

### RNA Extraction and rt-PCR

The cultured cells were rinsed with PBS twice, and the RNA was extracted by Trizol (Invitrogen). cDNA was synthesized using a FastQuant RT kit (TIANGEN, Beijing, China) according to the manufacturer’s instructions. The mRNA levels of genes were quantified by rt-PCR using LightCycler 480 SYBR Green I Master (Roche, Indianapolis, IN, United States) and a LightCycler 480 Roche System (Roche). The results were normalized against 18sRNA mRNA levels to determine the relative mRNA expression of genes. Sequences of the primers used for rt-PCR are listed in Table 2.

**TABLE 2:** List of siRNA sequences.

EGFR	Human	5′-GAGGAAATATGTACTACGA-3′
HMOX1	Human	5′-CAGCAACAAAGTGCAAGA-3′
Met	Human	5′-GAACAGAATCACTGACATA-3′
Nrf2	Human	5′-CAGTCTTCATTGCTACTAA-3′
VEGF-A	Human	5′-GGAGTACCCTGATGAGATC-3′
VEGF-C	Human	5′-CGACAAACACCTTCTTTAA-3′
VEGFR2 001	Human	5′-GGATGAACATTGTGAACGA-3′
VEGFR2 002	Human	5′- GGAGTGAGATGAAGAAATT-3′

### Western Blotting

The cultured cells were rinsed with cold PBS twice and lysed in RIPA buffer (Solarbio, Beijing, China) supplemented with complete protease and phosphatase inhibitors (Thermo Scientific, Waltham, MA, United States). The protein concentrations were measured utilizing a DC protein assay (Bio-Rad, Hercules, CA, United States). Next, 15 to 20 μg protein samples were loaded, separated on 8 or 15% sodium dodecyl sulfate-polyacrylamide gels and transferred to PVDF membranes. The membranes were blotted with 5% non-fat milk for 1 h at 25°C and probed with the following antibodies at 4°C overnight: rabbit anti-VEGFR2 (CST, #9698S, 1:1,000), rabbit anti-c-Met (CST, #8198, 1:1,000), rabbit anti-EGFR1 (CST, #2232, 1:1,000), rabbit anti-Bip (CST, #3177, 1:1,000), rabbit anti-IRE1α (CST, #3294S, 1:1,000), mouse anti-Chop (Abcam, #ab11419, 1:1,000), mouse anti-ATF6 (Santa Cruz, #sc-166659, 1:2,000), mouse anti-α-tubulin (Sigma, #T6074, 1:2,000), rabbit anti-Nrf2 (CST, #12721, 1:1,000), rabbit anti-HMOX1 (Abcam, #ab13248, 1:1,000), rabbit anti-p38 (CST, #8690, 1:1,000), rabbit anti-p-p38 (CST, #4511, 1:1,000), rabbit anti-PERK (CST, #5683, 1:1,000), rabbit anti-p-PERK (Abcam, #ab192591, 1:1,000), rabbit anti-eIF2 (CST, #5324, 1:1,000), rabbit anti-p-eIF2 (CST, #3398, 1:1,000), rabbit anti-JNK (CST, #9252, 1:1,000), rabbit anti-p-JNK (CST, #4668, 1:1,000), rabbit anti-IL-1β(CST, #12703, 1:1,000), rabbit anti-IL-6 (CST, #12153, 1:1,000), and goat anti-VEGF-A(R&D, #AF-293-NA, 1:500). The membranes were then washed three times by TBST and incubated with anti-rabbit, anti-mouse, or anti-goat horseradish peroxidase-conjugated antibodies (Liankebio, Hangzhou, China). Enhanced chemiluminescence kits (SuperSignal West Pico Chemiluminescent Substrate or SuperSignal West Femto Maximum Sensitivity Substrate, Thermo Scientific) were employed to detect the signals under the chemiluminescence imaging system, G-BOX (Syngene, Ballenger Creek, MD, United States).

### Cell Survival Assay

Cell survival was determined by counting and MTT assay. For cell counting, cells were initially seeded onto 6-well tissue culture plates (2 × 10^5^ cells/well, three replicates/group). Following treatments, the cells were trypsinized, and the cell suspensions were mixed with 0.4% trypan blue at a ratio of 1:1. The numbers of viable cells were counted using an automatic cell counter (CountStar, China). For MTT assay, cells were initially seeded onto 96-well tissue culture plates (1,000 cells/well, 6 replicates/group). After treatment, the cells in each well were incubated in MTT reagent (5 mg/ml, 10 μl/well) for 4 h at 37°C followed by medium removal. Then, 100 μl dimethyl sulfoxide was added to each well of the plates followed by vibration for 5 min. The absorbance at 570 nm was detected using a FLx800 Fluorescence Reader (Bio-tek, United States).

### Statistical Analysis

Statistical analyses were carried out using GraphPad Prism 5 software. For comparison of data between two groups, an unpaired two-tailed Student’s *t*-test was applied. For comparison between multiple groups, one-way ANOVA followed by Bonferroni’s multiple comparison test was performed. Results were represented as mean ± SD. Data were considered significant when *P* values < 0.05 (^∗^*P* < 0.05, ^∗∗^*P* < 0.01, ^∗∗∗^*P* < 0.001, and ^****^P < 0.0001), while they were not significant (N.S.) when *P* values ≥ 0.05.

## Results

### Glucose Depletion Resulted in Cell Death of RPE Cells and the Phenotype Was Reversed by Ablation of VEGFR2, but Not Its Ligands VEGF-A or VEGF-C

**FIGURE 1:**
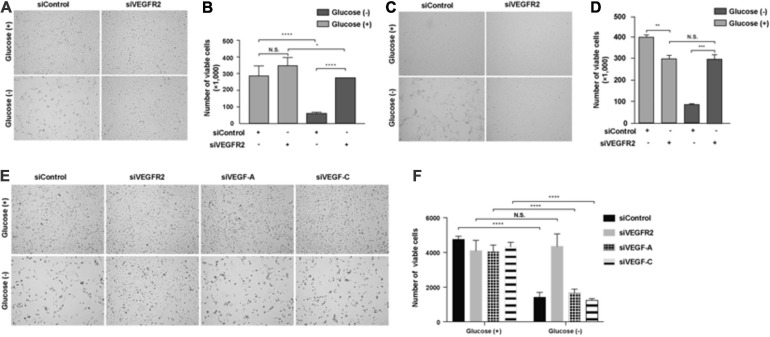
Depletion of VEGFR2 and not of its ligands VEGF-A or VEGF-C rescued glucose-depletion-mediated cell death in RPE cells. **(A)** Glucose depletion resulted in cell death, and VEGFR2 knockdown by siRNA rescued it in primary hRPE cells. **(B)** Quantification of **(A)**. **(C)** Glucose depletion resulted in cell death, and VEGFR2 knockdown rescued it in ARPE-19 cells. **(D)** Quantification of **(C)**. **(E)** Different from knockdown of VEGFR2, knockdown of VEGF-A and VEGF-C could not rescue the cell death caused by glucose depletion. **(F)** Quantification of **(E)**. Data in **(D,E)** are the means of three independent experiments (*n* = 3). All error bars indicate S.D. *P < 0.05, **P < 0.01, ***P < 0.001, ****P < 0.0001.

The retina is one of the most metabolically active tissues, and it mostly utilizes glucose as an energy source (Cohen and Noell, 1960). Glucose is transported from the choroidal blood supply to RPE cells first and then is delivered to photoreceptors (Wong-Riley, 2010). However, it remains unknown how RPE cells are affected by a lack of glucose. Additionally, the blockage of VEGF-A signaling has been utilized to treat wet AMD and other angiogenesis-related diseases of the eye. Thus, we decided to investigate the impacts of VEGF and its receptor VEGFR2 in RPE cells under glucose deprivation using siRNA knockdown. We found that the removal of glucose caused massive cell death of the primary RPE cells (Figure 1A left panel and Figure 1B). Surprisingly, efficient silencing of VEGFR2 by siRNA (Supplementary Figure 1A) reversed the phenotype (Figure 1A right panel and Figure 1B). A similar phenotype was reproduced in the immortalized ARPE-19 cell line in serum-free medium (Figures 1C,D). To eliminate the off-target issue, two siRNAs, designated as 001 and 002, were applied. Both could efficiently knock down VEGFR2 expression and rescue the cell death triggered by glucose deprivation. However, 002 provided more drastic results than 001 (Supplementary Figure 1). Thus, we selected 002 for subsequent experiments.

Since VEGF-A and VEGF-C, both of which are expressed in RPE cells (Zhao et al., 2006), serve as ligands to bind VEGFR2 and transduce signals (Simons et al., 2016), we studied if the depletion of either of them would result in a phenocopy knockdown of VEGFR2 under glucose starvation in RPE cells. Depleting neither VEGF-A nor VEGF-C reproduced the cell-death phenotype exhibited by the knockdown of VEGFR2, suggesting the VEGFR2 knockdown-mediated cell death was ligand-independent (Figures 1E,F). Taken together, these results indicated that the levels of VEGFR2 are critical for viability under glucose-deprived conditions.

### ER Stress-Related Signaling Pathways Were Changed by VEGFR2 Depletion

**FIGURE 2:**
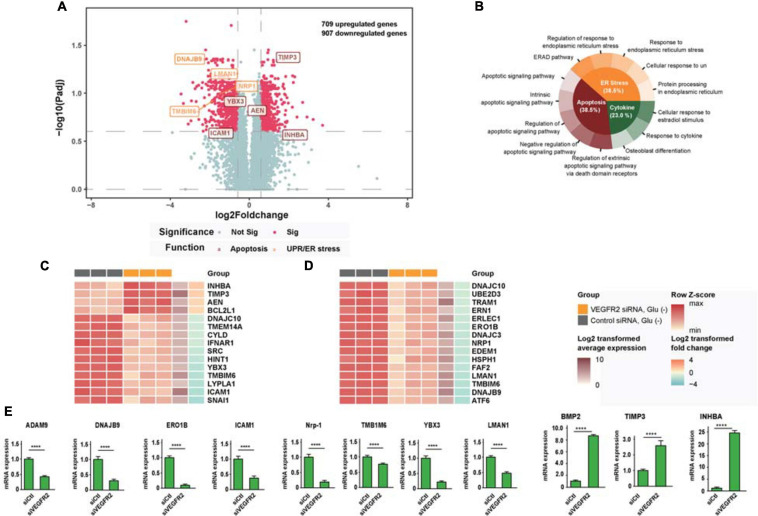
RNA-seq analysis links cell death rescue by deletion of VEGFR2 to ER stress and UPR-related signaling pathways upon glucose deprivation. **(A)** The volcano plot shows gene expression changes mediated by VEGFR2 knockdown, with ER stress and apoptosis genes highlighted. *X*-axis: log2 fold-change of gene expression between groups. *Y*-axis: -log10 adjusted *P* values of gene expression between groups. **(B)** GO and KEGG enrichment results showed that the biological terms affected by VEGFR2 knockdown were divided into three categories: apoptosis, ER stress, and cytokine. **(C)** Heatmap of differential gene expression in the apoptosis category. **(D)** Heatmap of differential gene expression in ER stress category. **(E)** Validation of differentially expressed genes by rt-PCR. **(A–E)** *n* = 3/group. All error bars indicate S.D. Unpaired student’s test, ****P < 0.0001.

To analyze the potential mechanisms underlying VEGFR2-associated cell death in RPE cells when exposed to glucose starvation, we carried out an RNA sequencing analysis to compare the genome-wide gene expression in primary RPE cells between VEGFR2 knockdown and the siRNA control. A total of 32,503 genes were detected, among which 709 genes were upregulated, whereas 907 genes were downregulated (Figure 2A). Further gene set enrichment analysis revealed that the most affected genes could be divided into three categories: Apoptosis, ER stress, and cytokine response (Figure 2B). Figure 2C revealed the differentially expressed genes involved in apoptosis, whereas Figure 2D showed the ones associated with ER stress and unfolded protein response. Several differentially expressed genes detected during the RNA sequencing could be validated via rt-PCR (Figure 2E). It is not surprising that apoptosis associated genes were detected since the data from Figure 1 showed that VEGFR2 knockdown rescued glucose-deprivation-mediated cell death. Terms associated with the typical genes of ER stress and unfolded protein response accounted for 38.5% of the total number of enriched biological terms, suggesting ER stress response and UPR were the underlying mechanisms responsible for the phenotype. Thus, we decided to focus on ER stress-related pathways in the subsequent experiments.

### VEGFR2 Is the Key Factor That Mediates ER Stress in RPE Cells Upon Glucose Deprivation

**FIGURE 3:**
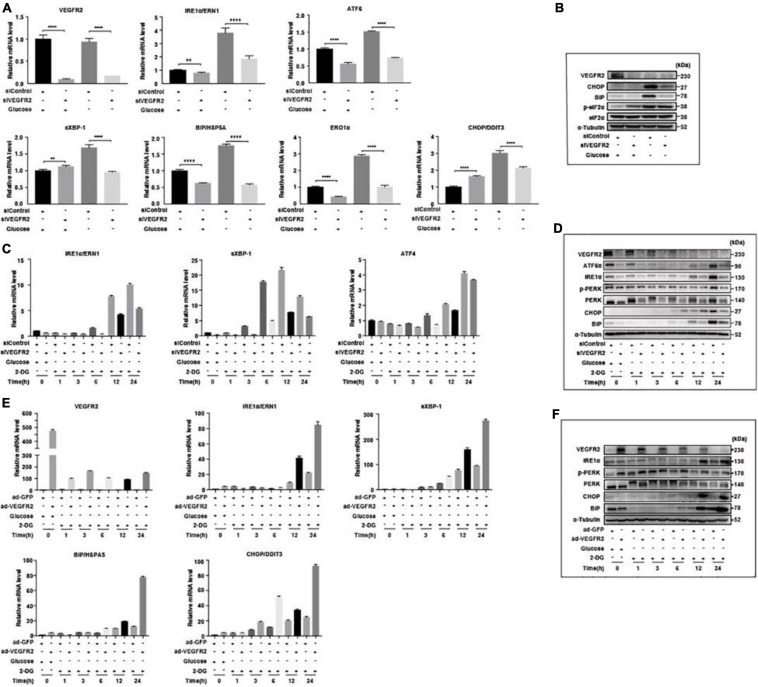
Level of VEGFR2 determined ER stress and UPR. **(A)** VEGFR2 knockdown via siRNA decreased glucose-depletion-mediated ER stress, as indicated by the UPR markers at the RNA level in primary hRPE cells. **(B)** VEGFR2 knockdown decreased the ER stress markers at the protein level in primary hRPE cells. **(C)** Time-course of the markers of glucose-depletion-mediated ER stress that was affected by VEGFR2 knockdown at the RNA level in ARPE-19 cells. **(D)** Time-course of the markers of glucose-depletion-mediated ER stress that was affected by VEGFR2 knockdown at the protein level in ARPE-19 cells. **(E)** Time-course of the markers of glucose-depletion-mediated ER stress that was affected by VEGFR2 overexpression at the RNA level in ARPE-19 cells. **(F)** Time-course of the markers of the glucose-depletion-mediated ER stress that was affected by VEGFR2 overexpression at the protein level in ARPE-19 cells. All error bars indicate S.D. **P < 0.01, ****P < 0.0001.

To confirm that ER stress-related pathways were indeed the underlying mechanism of the VEGFR2-associated phenotype under glucose starvation conditions, we knocked-down and overexpressed VEGFR2 in both hRPE and ARPE-19 cells cultured in glucose-free medium and then examined the ER stress markers. At the RNA level, ablation of VEGFR2 significantly decreased ER stress mediated by glucose deprivation, which was indicated by multiple markers including ERN1, ATF6, s-XBP1, HSPA5, ERO1α, and DDIT3 in hRPE cells (Figure 3A). The reduced ER stress was further validated at the protein level (Figure 3B). Similar results were observed in ARPE-19 cells (Figures 3C,D). Consistently, VEGFR2 overexpression in ARPE-19 cells drastically increased the markers of ER stress at both the RNA and protein levels (Figures 3E,F). Collectively, VEGFR2 was found to play a critical role in ER stress induction in the absence of glucose in RPE cells, and the cell death shown in Figure 1 is highly likely due to augmented ER stress.

### VEGFR2 Mediated ER Stress Is Not Only Limited to Glucose Deprivation

**FIGURE 4:**
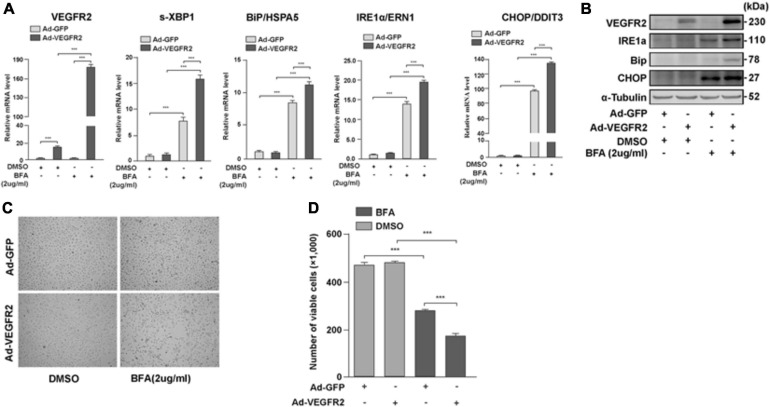
VEGFR2 mediated ER stress is not only limited to glucose deprivation. Overexpression of VEGFR2 further enhanced elevation of ER stress markers at the levels of RNA **(A)** and protein **(B)**, and increased cell death **(C)**. **(D)** Quantification of **(C)**. Data in **(D)** are the means of three independent experiments (*n* = 3). All error bars indicate S.D. ****P < 0.0001.

To test whether VEGFR2 mediated ER stress only through glucose-depletion, we overexpressed VEGFR2 in ARPE-19 cells followed by the treatment with an ER stress inducer, Brefeldin A (BFA), and measured the expression of ER stress markers. BFA inhibits the transportation of newly synthesized proteins from the ER to Golgi, resulting in accumulation of nascent peptides. As shown in Figures 4A,B, BFA unexpectedly increased the RNA and protein levels of overexpressed VEGFR2. Consequently, the markers of ER stress increased as expected at both the RNA and protein levels (Figures 4A,B). Consistently, cell death was increased in the VEGFR2 overexpressed cells compared to the control cells upon BFA treatment (Figures 4C,D). These results suggest again that VEGFR2 is essential for inducing ER stress, and that VEGFR2-mediated ER stress is a general phenomenon rather than being specific to glucose-depletion.

### VEGFR2 Is a More Potent ER Stress Inducer Under Glucose Deprivation Compared to Other Receptors

**FIGURE 5:**
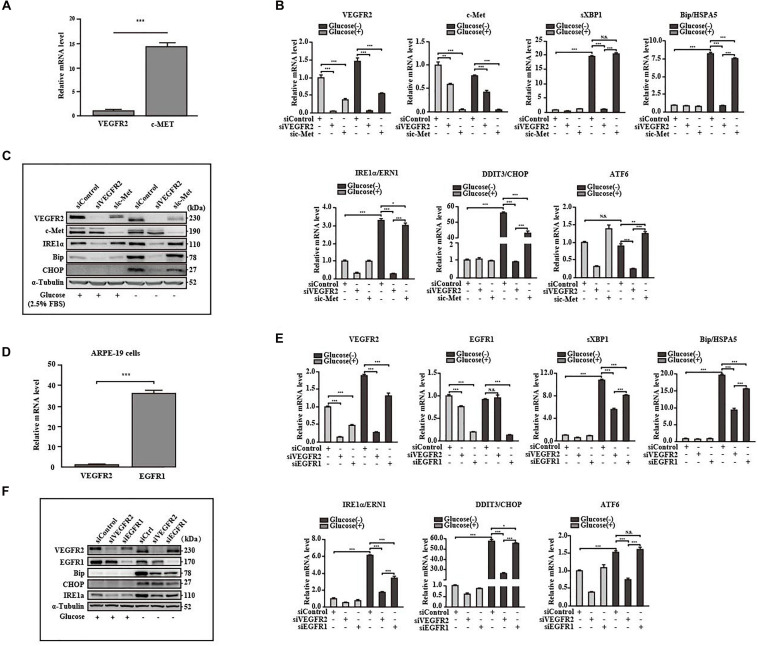
VEGFR2 was a potent ER stress inducer under glucose deprivation compared to other receptors. **(A)** Comparison of expression between VEGFR2 and c-Met at the RNA level. **(B,C)** Comparison of the ER stresses triggered by glucose deprivation between VEGFR2 and c-Met knockdown indicated by the markers at the RNA and protein levels, respectively. **(D)** Comparison of expression between VEGFR2 and EGFR1 at the RNA level. **(E,F)** Comparison of the ER stresses triggered by glucose deprivation between VEGFR2 and EGFR1 knockdown indicated by the markers at the RNA and protein levels, respectively. Data in **(A,B,D,E)** are the means of three independent experiments (*n* = 3). All error bars indicate S.D. *P < 0.05, **P < 0.01, ***P < 0.001.

Retinal pigment epithelium cells are known to also express EGFR1 (Yan et al., 2007) and c-Met (Cai et al., 2000), both of which are glycosylated (Stroop et al., 2000; Chen et al., 2013). We attempted to determine whether these receptors mediate ER stress responses to a similar degree as VEGFR2. Hence, we first compared the expression levels among the above receptors with that of VEGFR2. The levels of c-Met were 18 folds higher than those of VEGFR2 (Figure 5A). However, only one ER stress marker, DDIT3, was moderately reduced in comparison to the control in response to glucose-deprivation upon c-Met ablation at both the RNA and the protein levels (Figures 5B,C). These results suggest that c-Met fails to induce ER stress to a similar degree as VEGFR2. The RNA level of EGFR1, a well-known receptor expressed in RPE cells, was 40 times higher than that of VEGFR2, suggesting that the abundance of EGFR1 was significantly higher than that of VEGFR2 (Figure 5D). Nevertheless, although up to 90% of EGFR1 knockdown alleviated glucose-deprivation induced ER stress (Figures 5E,F), the alleviation was much less compared to the VEGFR2 knockdown particularly at the RNA level (Figure 5E) and to some degrees, at the protein level (Figure 5F). Interestingly, VEGFR2 silencing resulted in a reduction of EGFR1 under both glucose and glucose-free conditions, whereas the knockdown of EGFR1 decreased the level of VEGFR2 only under glucose condition (Figure 5C), which may explain the difference between the two receptors in terms of triggering ER stress. Taken together, these results suggest that even though VEGFR2 is not an abundant glycosylated receptor, it has strong capacity to induce ER stress under glucose-deprivation conditions in RPE cells.

### VEGFR2 Knockdown Lowered Nrf2 Signaling Pathway

**FIGURE 6:**
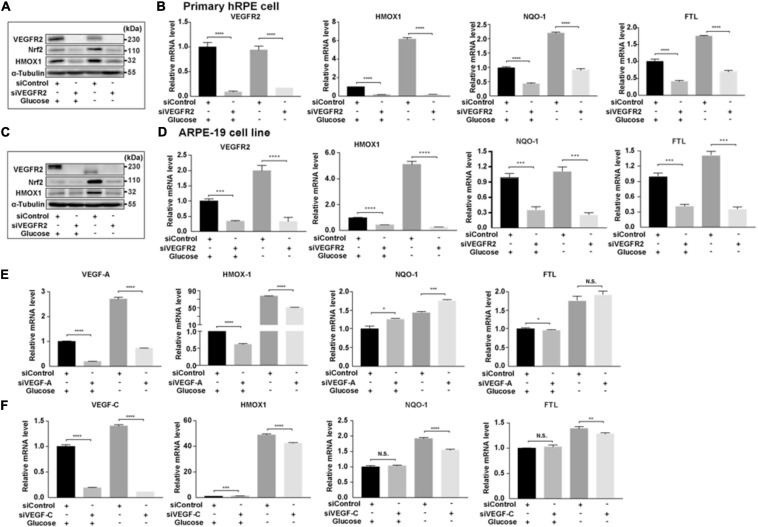
Depletion of VEGFR2 alleviated Nrf2 signaling pathway. **(A)** VEGFR2 knockdown via siRNA reduced glucose-deprivation-mediated Nrf2 protein accumulation and its downstream target HMOX-1 in primary hRPE cells. **(B)** VEGFR2 siRNA knockdown decreased the expression of Nrf2 downstream targets in primary hRPE cells. **(C)** VEGFR2 siRNA knockdown decreased Nrf2 and HMOX-1 protein accumulation in ARPE-19 cells. **(D)** VEGFR2 siRNA knockdown decreased the expression of Nrf2 downstream targets in ARPE-19 cells. **(E)** VEGF-A siRNA knockdown was unable to reduce glucose-deprivation-mediated gene expression of Nrf2 downstream targets. **(F)** VEGF-C siRNA knockdown was unable to reduce glucose-deprivation-mediated gene expression of Nrf2 downstream targets. All error bars indicate S.D. *P < 0.05, **P < 0.01, ***P < 0.001, ****P < 0.0001.

One of the downstream targets of ER stress is Nrf2 (Oakes, 2020), which responds to increased oxidative stress (Ma, 2013). To determine whether the signal mediated by the changed VEGFR2 levels passed from ER stress to its downstream, we examined the expression of Nrf2 and its downstream target genes. In primary hRPE cells, while glucose depletion increased the protein level of Nrf2 as expected, VEGFR2 knockdown significantly reduced Nrf2 expression (Figure 6A). HMOX1, a well-known Nrf2 target gene, exhibited the same pattern. At the RNA level, HMOX1 and two other Nrf2 target genes, NQO-1 and FTL, were also expressed in a similar manner (Figure 6B). Similar results were observed in ARPE-19 cells (Figures 6C,D). In Figure 1, knockdown of VEGF-A and VEGF-C could not mimic the rescue of cell death by VEGFR2 knockdown upon glucose depletion. As expected, knockdown of VEGF-A and VEGF-C could not decrease the glucose-depletion-mediated elevation of Nrf2 target genes including HMOX1, NQO-1, and FTL (Figures 6E,F). Together, these results suggest that a decrease in VEGFR2 can reduce the glucose-depletion-mediated Nrf2 signaling via a diminished ER stress response.

### VEGFR2 Knockdown Resulted in Altered Expression of Inflammatory Factors

**FIGURE 7:**
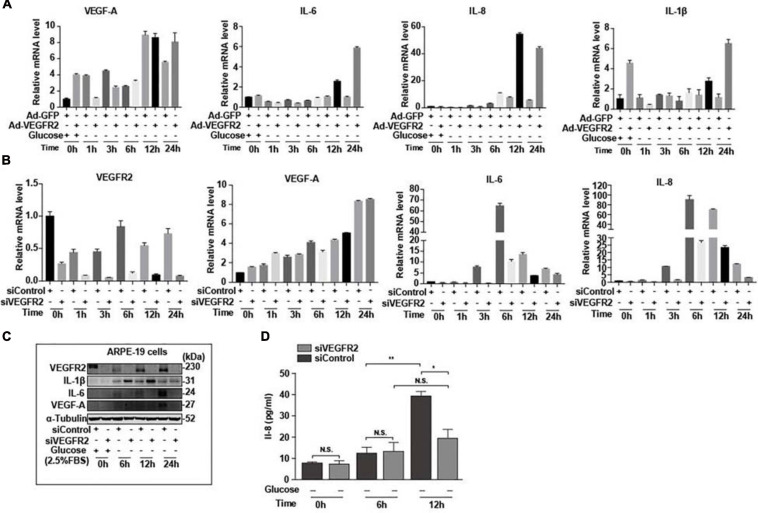
Level of VEGFR2 determined inflammation under glucose deprivation. **(A)** Overexpression of VEGFR2 increased expression of inflammatory factors at the RNA level. **(B)** VEGFR2 knockdown decreased expression of inflammatory factors at the RNA level. **(C)** VEGFR2 knockdown changed expression of inflammatory factors at the protein levels. **(D)** VEGFR2 knockdown decreased IL-8 expression measured by ELISA. All error bars indicate S.D. *P < 0.05, **P < 0.01, ***P < 0.001, ****P < 0.0001.

Endoplasmic reticulum stress affects not only the oxidative stress via Nrf2 but also inflammation through multiple pathways (Oakes, 2020). Furthermore, RPE-mediated inflammation is one of the major causes of AMD (Perez and Caspi, 2015). To determine the impact of VEGFR2 levels on inflammation in RPE cells upon glucose removal, VEGFR2 was knocked-down and overexpressed in ARPE-19 cells, and the levels of various inflammatory factors were evaluated. Overexpressed VEGFR2 upregulated typical inflammatory factors, including IL-6, IL-1β, and IL-8, at the RNA level, whereas the expression of VEGF-A was not affected even though its expression was induced by glucose deprivation (Figure 7A). Consistently, VEGFR2 knockdown downregulated the expression of IL-6 and IL-1β but not of VEGF-A at the RNA level (Figure 7B). However, at the protein level, VEGFR2 knockdown considerably downregulated the expression of VEGF-A together with IL-6. In contrast, the protein level of IL-1β increased with VEGFR2 knockdown (Figure 7C). In addition, an ELISA assay indicated that VEGFR2 knockdown reduced the IL-8 protein level (Figure 7D). These results suggest that the VEGFR2 level influences inflammatory factors at multiple levels and affects different factors in a different manner.

## Discussion

**FIGURE 8:**
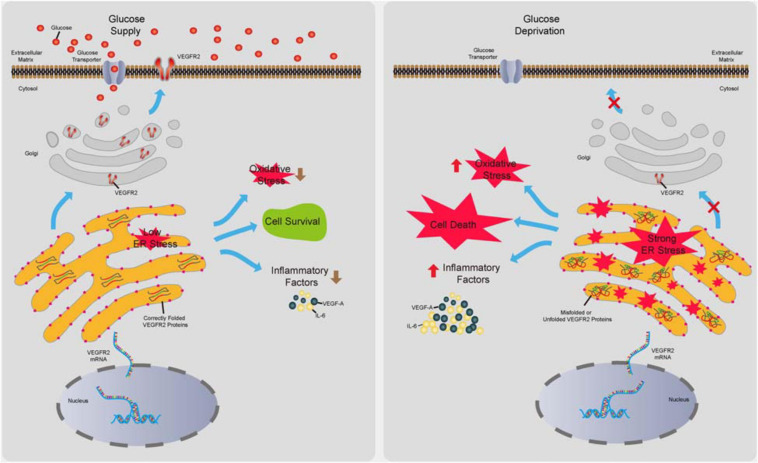
Summary of the novel role of VEGFR2 identified in the current study. In the presence of glucose, VEGFR2 is translated, glycosylated, and folded correctly in the ER lumen and is transported to the cell membrane. Consequently, a normal level of ER stress is maintained, resulting in cell survival and lower levels of oxidative stress and inflammatory factor expression. Under glucose deprivation, under-glycosylated VEGFR2 is incorrectly folded and trapped in the ER, which gives rise to a higher level of ER stress. Expectedly, it triggers cell death and increased oxidative stress and inflammatory factor expression.

Retinal pigment epithelium dysfunction is associated with several eye diseases including AMD, which is classified into dry AMD and wet AMD. The former displays loss of RPE cells, while the latter is characterized by outgrowth of choroidal blood vessels penetrating RPE and the Bruch’s membrane mostly due to abnormal secretion of proangiogenic factors from RPE cells (Lipecz et al., 2019). Even though wet AMD is routinely treated via anti-VEGF-A based therapy (Yazdi et al., 2015), this therapy can only delay the progress of the disease due to a lack of detailed understanding of how the proangiogenic factors are induced. In contrast, no efficacious drugs for dry AMD are currently available. Thus, the mechanisms of how RPE cells maintain their functions to avoid cell death and overproduce proangiogenic factors need to be elucidated to prevent or delay the pathogenesis of AMD. Evidence has indicated that function loss and morphological changes of choriocapillaris, which supplies oxygen and glucose as well as other nutrients to RPE and photoreceptors, occur at various stages of AMD (Thulliez et al., 2019) preceding RPE degeneration (Alagorie et al., 2019). These studies suggest the existence of ischemia during AMD initiation. Although recent studies have shown that hypoxia in RPE could result in AMD phenotypes in mouse models (Kurihara et al., 2016), the effects of glucose deprivation on RPE remain to be investigated. Furthermore, as a major drug target for anti-angiogenesis therapy, VEGF-A and its associated signaling in endothelial cells have been the focus of many studies (Simons et al., 2016); however, the autocrine VEGF-A/VEGFR2 axis in RPE cells has received little attention. In this study, we attempted to identify the impacts of VEGFR2 in RPE cell survival, oxidative stress, and inflammation under glucose deprivation. We found that the level of VEGFR2 was a key factor for determining cell survival under glucose starvation. The underlying mechanism could be associated with the overwhelming ER stress caused by unfolded VEGFR2, resulting from insufficient glycosylation. Interestingly, even though the VEGFR2 levels were much lower compared to those of EGFR1 and c-Met, the other two glycosylated receptors in RPE cells, changes in VEGFR2 levels affected the ER stress much more potently. Downregulation of VEGFR2 resulted in lower oxidative stress, as indicated by reduced Nrf2 signaling as well as decreased expression of inflammatory factors including VEGF-A, IL-6, and IL-8. These results suggest that the roles of VEGFR2 can be ambivalent depending on the glucose supply. Under physiological conditions with normal blood flow to supply glucose, VEGFR2 serves as a receptor to transduce prosurvival signals by binding to VEGF-A (Ford et al., 2011). Under ischemic conditions in which glucose supply is scarce, a high level of VEGFR2 triggers strong ER stress, leading to cell death due to incomplete glycosylation of the VEGFR2 peptide (summarized in Figure 8). Thus, to the best of our knowledge, our current study, for the first time, demonstrates that VEGFR2 can exert detrimental effects on RPE cells, which is contrasting to the conventional perception.

VEGFR2 is a highly glycosylated protein (Chandler et al., 2017). Previous studies have reported that decreasing its glycosylation can lead to its degradation in cancer cells (Adham and Coomber, 2009) and endothelial cells (Kovacs et al., 2016). The latter group showed that glucose deprivation by 2-DG triggered apoptosis in endothelial cells. Furthermore, the authors argued that the diminished VEGF signaling due to VEGFR2 degradation was responsible for cell death (Kovacs et al., 2016). We also observed the slower mobility-shifted VEGFR2 band in the western blotting assay, and its abundance decreased upon glucose deprivation, suggesting a similar mechanism regarding VEGFR2 degradation occurs in RPE cells. However, according to the robust evidence present in the current study, we are convinced that excessive ER stress itself is an inducer of cell death and the level of VEGFR2 peptide is a key determinant for ER stress, which, to the best of our knowledge, has not been reported thus far.

Although it is known that RPE cell death occurs in geographic atrophy (GA) and changes in secretion of factors from RPE cells including VEGF-A, in turn, causes CNV in wet AMD (Ambati and Fowler, 2012), how early AMD gradually advances into these late stages is not completely understood. Furthermore, the reasons why some early AMD cases progress into GA status while the others evolve with CNV lesion are also unclear. In the current study, we revealed that ER stress can be at least one of the hubs that play critical roles in both dry and wet AMD. When ER stress accumulates to such a degree that cells cannot handle the stress, cells will undergo apoptosis (Cybulsky, 2017). Our current study revealed that, at least *in vitro*, glucose starvation resulted in massive cell death, indicating that *in vivo* ischemia induced by choriocapillaris degeneration may potentially lead to RPE cell death and thus the onset of dry AMD. Since VEGF-A from RPE cells plays a critical role in supporting choriocapillaris (Saint-Geniez et al., 2009), the death of local RPE cells may promote further choriocapillaris degeneration, thereby forming a vicious cycle that exacerbates the disease. Our results also showed that cell death induced by glucose deprivation can be context dependent. Cell death could be triggered by a lack of glucose in primary hRPE cells even in the presence of 10% FBS, whereas it occurred in ARPE-19 cell when FBS was below 2.5%. Thus, if the RPE cells survived glucose starvation, an increase in ER stress can lead to elevated inflammation and secretion of proangiogenic factors such as VEGF-A and IL-6 (Ghasemi, 2018), which may be accountable for the progression of wet AMD. Therefore, the control of ER stress by manipulating VEGFR2 levels may provide an option to prevent or delay the pathogenesis of both dry and wet AMD.

Given that N-linked glycosylation is essential for nascent peptides to fold correctly in ER lumen, interruption of the HBP pathway by glucose deprivation can lead to accumulation of any unfolded or misfolded glycosylated proteins, thus resulting in ER stress. As mentioned before, ER stress activates UPR, leading to the degradation of those proteins. Most growth factor receptors are located on the cell membrane and are glycosylated; results of our study showed that the capacity of the receptors in inducing ER stress does not have a positive correlation with their expression levels. For instance, the expression of VEGFR2 is much lower than that of EGFR1 and c-Met; however, VEGFR2 appears to trigger ER stress to a much higher degree even though all of them displayed degradation upon glucose deprivation. Other studies also showed that in cancer cells, VEGFR1, also a glycosylated receptor binding VEGF-A (Bruns et al., 2010), did not undergo degradation upon glucose starvation (Adham and Coomber, 2009). Furthermore, Bruns et al. reported that VEGF-A binding triggered the degradation of VEGFR2 but not of VEGFR1 (Bruns et al., 2010). These results suggest that VEGFR2 is a unique protein in terms of its stability and roles in promoting cell death or survival.

In summary, our current study has identified a novel effect of VEGFR2, which is a potent capacity to mediate ER stress under glucose depletion which can trigger cell death. This contrasts with its role in promoting cell survival under physiological conditions with sufficient glucose supply. Consistently, oxidative stress and the secretion of proangiogenic factors such as VEGF-A and IL-8 were also controlled by the level of VEGFR2 via ER stress and the corresponding UPR signaling. Our results provide an insight into the mechanisms of AMD pathogenesis and suggest that VEGFR2 can be applied as a novel therapeutic target to prevent AMD initiation or impede the progression of the disease. Further *in vivo* investigations will be help to achieve such a goal.

## Data Availability Statement

The datasets presented in this study can be found in online repositories. The names of the repository/repositories and accession number(s) can be found below: NCBI GEO GSE1 64167 https://www.ncbi.nlm.nih.gov/geo/query/acc.cgi?acc= GSE164167.

## Author Contributions

WC, XL, and RJ designed and directed the project. BX, LZ, QC, LH, ZY, and XR performed the experiments. JZ, BX, and LZ processed the data and carried out the data analysis. SW and RJ supervised the project. YC and LJ aided in interpreting the results and worked on the manuscript. All the authors discussed the results and commented on the manuscript.

## Conflict of Interest

The authors declare that the research was conducted in the absence of any commercial or financial relationships that could be construed as a potential conflict of interest.
