# Supplementary figures and images for: Role of VEGFR2 in Mediating Endoplasmic Reticulum Stress Under Glucose Deprivation and Determining Cell Death, Oxidative Stress, and Inflammatory Factor Expression
Source: Front Cell Dev Biol. 2021 Jun 18;9:631413. doi: 10.3389/fcell.2021.631413 (PMC8249873; doi:10.3389/fcell.2021.631413)

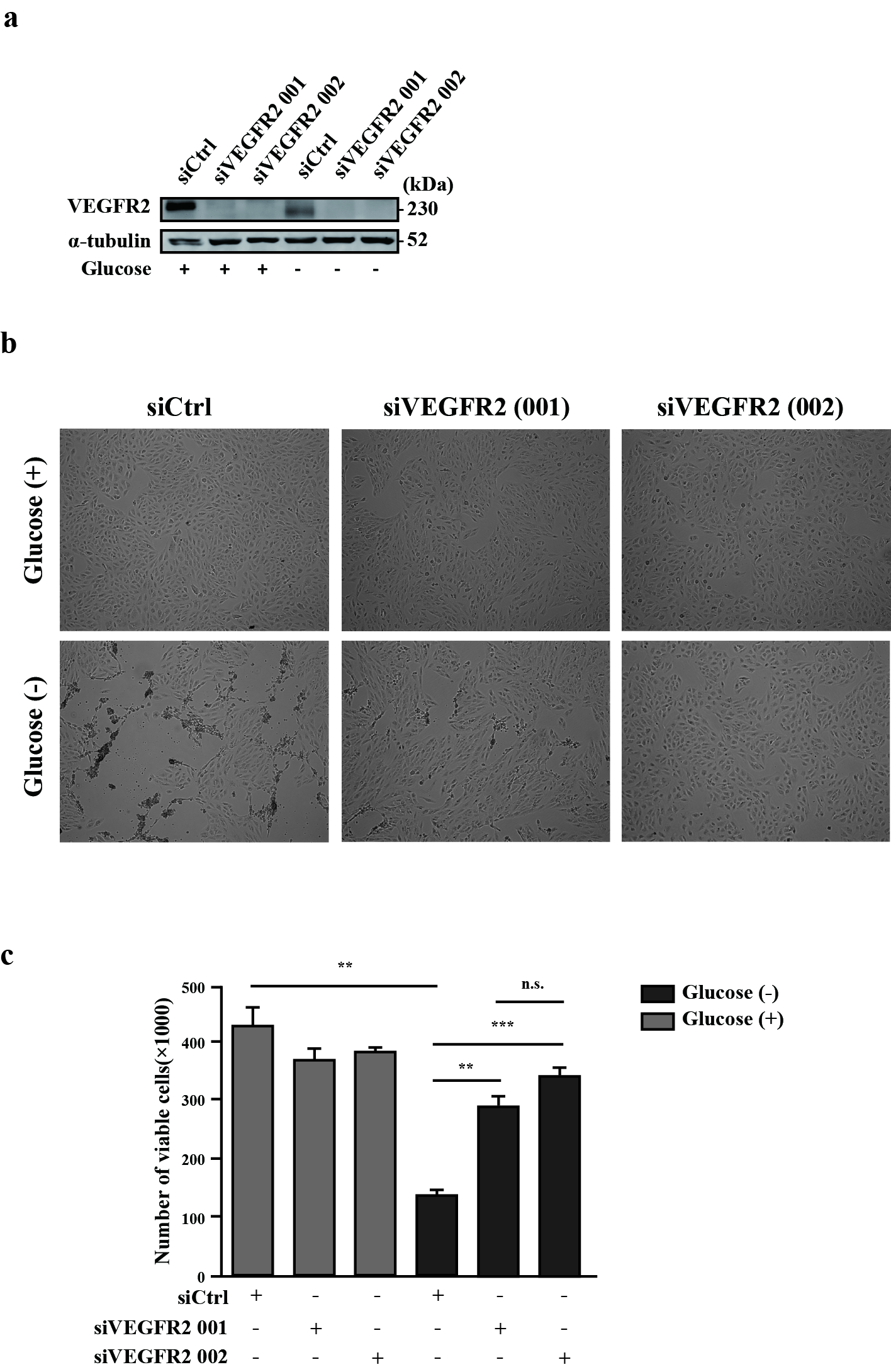

Supplement: Supplementary file 1 [file Image_1.TIF]
